# Corazonin Signaling Is Required in the Male for Sperm Transfer in the Oriental Fruit Fly *Bactrocera dorsalis*

**DOI:** 10.3389/fphys.2018.00660

**Published:** 2018-06-04

**Authors:** Qiu-Li Hou, Er-Hu Chen, Hong-Bo Jiang, Shuai-Feng Yu, Pei-Jin Yang, Xiao-Qiang Liu, Yoonseong Park, Jin-Jun Wang, Guy Smagghe

**Affiliations:** ^1^Key Laboratory of Entomology and Pest Control Engineering, College of Plant Protection, Southwest University, Chongqing, China; ^2^Academy of Agricultural Sciences, Southwest University, Chongqing, China; ^3^Department of Entomology, Kansas State University, Manhattan, KS, United States; ^4^Department of Plants and Crops, Ghent University, Ghent, Belgium

**Keywords:** corazonin, male adult, reproduction, sperm transfer, mating duration

## Abstract

Corazonin (Crz) is a widely distributed neuropeptide (or neurohormone) in insects with diverse physiological functions. The present study aimed to reveal the functions of Crz and its receptor (CrzR) in the regulation of sexual behavior and fertility in male *Bactrocera dorsalis*. Tissue-specific expression analyses showed that the *BdCrz* transcript was most abundant in the central nervous system (CNS), and the *BdCrzR* transcript was most abundant in both the fat body and CNS. Immunochemical localization confirmed that three pairs of Crz-immunoreactive neurons are located in the dorsolateral protocerebrum region of male adult brain. Importantly, RNAi-mediated Crz knockdown lengthened mating duration in males, and knockdown of Crz or CrzR strongly decreased male fertility in the following 3 days, while the courtship behavior and mating efficiency were not affected. The reduced number of sperm in the reproductive organs of mated females indicated that Crz knockdown in males reduced sperm transfer. The findings of this study indicate that Crz contributes to the reproductive physiology of the oriental fruit fly *B. dorsalis* by regulating sperm transfer in male adults.

## Introduction

The oriental fruit fly, *Bactrocera dorsalis* (Hendel) (Diptera: Tephritidae), is a widely distributed quarantine polyphagous pest in tropical and subtropical areas of the world. Due to the increased invasiveness and potential influence on food production, *B. dorsalis* has been one of the most destructive pest of fruit industries in many countries (Clarke et al., 2005; Stephens et al., 2007). Because of the rapid development of insecticide resistance and particular way of damage, this insect became resurgence and difficult to control (Chen et al., 2013; Huang et al., 2015), and therefore, new insecticide targets are urgently needed. Insect neuropeptide signaling systems have been regarded as a novel type of pest management (Altstein, 2001; Boonen et al., 2009; Altstein and Nässel, 2010).

Insect neuropeptides are essential physiological regulators for various biological events such as development, metabolism, and behavior. They also play a key role in the regulation of sexual behavior and reproduction (Altstein and Nässel, 2010). The peptide corazonin (Crz) was first identified as a cardioactive peptide in the American cockroach *Periplaneta americana* (Veenstra, 1989). Subsequent identification of the Crz in other insect species revealed two major members of the Crz family: [Arg^7^]-Crz and [His^7^]-Crz (Lee et al., 2008). In our previous study, we have identified and characterized the [His^7^, Ser^11^]-Crz isoform (pQTFQYSHGWTSamide) in *B. dorsalis* (Hou et al., 2017b), which is different from the [Arg^7^]-Crz (pQTFQYSRGWTNamide) as reported in several *Drosophila* species (Veenstra, 1994; Choi et al., 2005), and also different isoforms in other insects have been reported so far (Predel et al., 2007; Hou et al., 2017b). The Crz receptor (CrzR) of *B. dorsalis* is a typical Class A GPCR member with seven transmembrane domains, showing a high sequence similarity to the Crz receptor in *Drosophila* (*DmCrzR*) (Hou et al., 2017b).

Molecular and histochemical studies have demonstrated that the distribution of Crz in neurosecretory neurons of the central nervous system (CNS) seems to be conserved among different insect species (Predel et al., 2007; Lee et al., 2008). Various functions have been found for physiological and behavioral actions in different experimental systems adopted in diverse insect groups. For example, a significant role for Crz in modulating myostimulatory activities on the heart muscle of the cockroach *P. americana* (Veenstra, 1989) and the hyperneural muscle of stick insect *Carausius morosus* (Predel et al., 1999), while the myostimulatory activity of Crz was not found in other insects (Predel et al., 1994; Sláma et al., 2006; Hillyer et al., 2012). Physiological studies with the moth *Manduca sexta* suggested that Crz signaling initiated the ecdysis behavior by inducing the release of pre-ecdysis and ecdysis-triggering hormones from the Inka cells (Kim et al., 2004). In *D. melanogaster*, ablation of Crz neurons affected the metabolism and lead to a differential lifespan under stress, which showed a strong dependence on sex (Zhao et al., 2010). More importantly, silencing of Crz neurons affected the mating behavior and blocked the transfer of sperm and seminal fluid in *D. melanogaster* (Tayler et al., 2012). Peptide injection to the early transitioning ants demonstrated that Crz stimulates hunting and inhibits dueling in the ant *Harpegnathos saltator* (Gospocic et al., 2017). Crz and CrzR have also been identified using a heterologous functional assay in *Aedes aegypti* (Oryan et al., 2018). In our earlier study, the silencing of CrzR by RNAi effectively disrupted the larval-pupal transition and led to a delay in pupariation in *B. dorsalis* (Hou et al., 2017b). Crz-type signaling is also present in other invertebrates, including other protostomes (annelids and mollusks) and deuterostomes (cephalochordates, hemichordates, and echinoderms) (Zandawala et al., 2017). For example, Kavanaugh and Tsai (2016) have characterized a Crz-type signaling system in *Aplysia* and interestingly the Crz-type receptor is expressed in reproductive organs (small hermaphroditic duct, and ovotestis). A Crz-type signaling system has also been characterized in the starfish *Asterias rubens*, which causes contraction of cardiac stomach, apical muscle, and tube foot preparations (Tian et al., 2016, 2017). Another recent publication showed, however, that Crz had no effect on heart activity, blood glucose levels, lipid mobilization, or pigment distribution in chromatophores in the crab *Carcinus maenas* (Alexander et al., 2017).

To gain further insight into the function of Crz in *B. dorsalis*, in the present study, we have extended our previous work with the larval stages (Hou et al., 2017b) now with the adult stage to investigate the sexual behavior regulation of the Crz/CrzR signaling pathway by RNAi. Our data may contribute to explore its potential as a novel insecticide target.

## Materials and Methods

### Test Insects

*Bactrocera dorsalis* was originally collected from Dongguan, Guangdong province, China, and reared as described previously (Chen et al., 2013). Shortly, the flies were reared at 27 ± 1°C, 70 ± 5% relative humidity with a photoperiod of 14 h light: 10 h dark. Shortly after the adult emergence, virgin flies sexed and kept isolated on a standard medium until they were used for experiments. In this study, we used the 9-day-old virgin flies in most experiments.

### Sample Preparation, RNA Isolation, and cDNA Synthesis

Tissues from the CNS, fat body, midgut, hindgut, Malpighian tubules, and testis were excised from 9-day-old adults to determine the tissue-specific expression pattern. At least 15 individuals were dissected as one sample for tissue-specific analysis with four replications. The adults were chilled on ice for 30 min and dissected under a stereomicroscope (Olympus SZX12, Tokyo, Japan). The samples were isolated on ice, placed in a 2.0 mL of diethyl pyrocarbonate (DEPC)-treated centrifuge tube containing RNA storage reagent (Tiangen, Beijing, China), and immediately frozen in liquid nitrogen and stored at -80°C.

Total RNA was extracted from each sample using the TRIzol reagent (Invitrogen, Carlsbad, CA, United States) and treated with DNase I (Promega, Madison, WI, United States) to prevent potential genomic DNA contamination. First-strand cDNA was synthesized using the GoScript Reverse Transcription System (Promega) according to the manufacturer’s instructions.

### Quantitative Real-Time PCR (qPCR)

Primers used in this study (**Table 1**) were synthesized by Invitrogen (Shanghai, China). qPCR was performed on an ABI 7500 Real-Time PCR System (Applied Biosystems, Foster City, CA, United States). The reaction mixtures: cDNA template (0.5 μL), of each primer (0.5 μL, 0.2 mM), Novostar-SYBR Supermix (5 μL, Novoprotein, Shanghai, China) and of ddH_2_O (3.5 μL). Reaction conditions: an initial denaturation at 95°C for 2 min, followed by 40 cycles of 95°C for 15 s, 60°C for 30 s. At the end of each qPCR, a melting curve analysis from 60 to 95°C was generated to rule out the possibility of primer-dimer formation. The data were normalized to the stable reference gene *α-tubulin* (GenBank accession no. GU269902) based on previous evaluations (Shen et al., 2010). The relative expression levels were calculated using the 2^-ΔΔCt^ method (Livak and Schmittgen, 2001).

**Table 1:** Primer sequences used in this study.

Target	Direction	Sequence 5′ to 3′
*BdCrz* (qPCR)	Forward	TTGCCGAAATGCTCCAACAA
	Reverse	CCATAATCGTTCGTCTCGGC
*BdCrzR* (qPCR)	Forward	TGCTCACCGTCACCTACATT
	Reverse	TCACAAAAGACAGTCGCAGC
*α-Tubulin* (qPCR)	Forward	CGCATTCATGGTTGATAACG
	Reverse	GGGCACCAAGTTAGTCTGGA
*BdCrz* (dsRNA)	Forward	taatacgactcactatagggTCTTCTCATTGGCTGTCCTCT
	Reverse	taatacgactcactatagggTAGATTACCATTTGCTGGAGTCAG
*BdCrzR* (dsRNA)	Forward	taatacgactcactatagggTTACACAAATCGACGGCAGC
	Reverse	taatacgactcactatagggGCTGTGTGAATTTGCATCGC
GFP (dsRNA)	Forward	taatacgactcactatagggCAGTTCTTGTTGAATTAGATG
	Reverse	taatacgactcactatagggTTTGGTTTGTCTCCCATGATG


### Immunohistochemistry

For immunohistochemical detection of Crz in *B. dorsalis*, we used the polyclonal Crz antibody (against *D. melanogaster* Crz, pQTFQYSRGWTNamide) that was a gift from Dr. Jan Veenstra (Université de Bordeaux, France; Veenstra and Davis, 1993) with use of our protocol as previously published (Hou et al., 2017b). In brief, the brains from 9-day old males were dissected in chilled PBS (pH 7.4) and then tissues were fixed overnight at 4°C in fresh 4% paraformaldehyde in PBS. After washing for 3×5 min in PBS with 0.5% Triton X-100 (PBST), the tissues were incubated with primary antibody (1:1000 diluted in PBST) for 2 days at 4°C and then washed in 3×5 min in PBST. Tissues were incubated overnight at 4°C in Alexa 488-conjugated goat anti-rabbit IgG antibody (1:1000 in PBST). The samples were washed in 2×5 min in PBST. Then, samples were mounted on a clean slide with 100% glycerol. Images were captured in a confocal microscope Zeiss LSM780 (Zeiss, Jena, Germany).

### RNAi Bioassay

Primers (**Table 1**) with T7 promoter sequences were used to amplify a fragment of *BdCrz*, *BdCrzR*, or *GFP* (CAA58789) for the double-stranded RNA (dsRNA) synthesis. The dsRNA was transcribed by Transcript Aid T7 High Yield Transcription Kit (Thermo Scientific, Lithuania) following the manufacturer’s protocol. The dsRNA was quantified using a NanoPhotometer (Implen, Germany) and the integrity was confirmed by a 1% agarose gel electrophoresis.

For the RNAi experiments, 9-day-old adult virgin flies were used. Then 1.2 μg (in 300 nL) of dsRNA (Crz-dsRNA, CrzR-dsRNA or GFP-dsRNA) was injected into the body cavity of the 9-day-old males directly between the second and third abdominal segments using a Nanoject II Auto-Nanoliter Injector (Drummond Scientific, Broomall, PA, United States). Injected flies were kept on an artificial diet under the conditions described above. The RNAi efficiencies of Crz and CrzR were measured by qPCR as described above, and analyzed by an independent Student’s *t*-test.

### Behavioral Assays

The mating behavior assays were performed as in our previous report (Hou et al., 2017a). All assays were performed in the mating arenas (a small round inverted cup with 6 cm in diameter and 10 cm high) under standard laboratory conditions. Virgin females and native males were collected separately on the day of adult emergence. Subsequently, 9-day-old male flies were injected with dsRNA. Twenty-four hours after the dsRNA injection, the treated males were crossed to the normal females individually and reared in the mating arenas. Each pair of flies was transferred to the mating chamber at the beginning of the light photoperiod to ensure adequate circadian time for acclimation. Mating behavior was observed every 30 min over a 10 h-dark photoperiod or until the male dismounted the female following mating. For the behavior assay, 30 pairs of flies were used per treatment with three biological replicates. Courtship behavioral sequence was based on the previous reports (Poramarcom, 1988), which is including the steps from wing vibration to attempting copulation. The percentage of males displaying courtship behavior, the percentage of mating pairs and the copulation duration were recorded. Furthermore, the numbers of eggs laid by successfully mated flies were recorded at 24 h-intervals for 3 days following mating.

### DAPI Straining

After the completion of mating behavior, the reproductive organs (spermatheca) of mated females were dissected in PBS. Then the tissues were gently grinded and stained with DAPI (4′,6-diamidino-2-phenylindole, 2 μg/ml) (Sigma, St. Louis, MO, United States) for 15 min. A fluorescence microscope Zeiss LSM780 (Zeiss, Jena, Germany) was used for images capture and cells counting.

### Statistical Analysis

Data on copulation durations were analyzed by a non-parametric Mann–Whitney *U* test, and these on mating and fertility by an independent Student’s *t*-test or one-way ANOVA, both in SPSS 16.0 software (SPSS, Chicago, IL, United States, 2008). Results are presented as mean ± SE (standard error), and statistical significance is assumed for *p* < 0.05.

## Results

### Tissue-Specific Transcript Profiling

**FIGURE 1:**
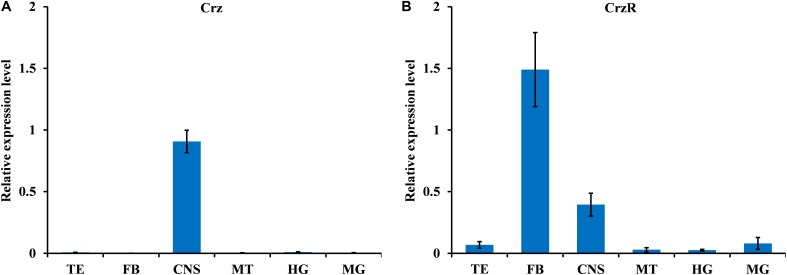
Relative expression levels of *BdCrz* **(A)** and *BdCrzR* **(B)** in different tissues of the 9-day-old male adult of *B. dorsalis*. Abbreviations used on the *X*-axis: TE, testis; FB, fat body; CNS, central nervous system; MT, malpighian tubules; HG, hindgut; MG, midgut. The expression levels were normalized against *α-tubulin* as internal reference gene and shown as relative expression levels. Data are means ± SE based on four independent experiments.

We analyzed the spatial distribution of *BdCrz* and *BdCrzR* in different organs of the adult by qPCR. As shown in **Figure 1**, *BdCrz* was mainly expressed in the CNS, and no detectable qPCR products were determined in the other tissues tested (as the *C*t values with the other tissues were >35) (**Figure 1A**). The transcript of *BdCrzR* was highly accumulated in the fat body, and at medium levels in the CNS. Low-level expressions were observed in testis, malpighian tubules, and gut (**Figure 1B**).

### Localization of *BdCrz* in the Brain

**FIGURE 2:**
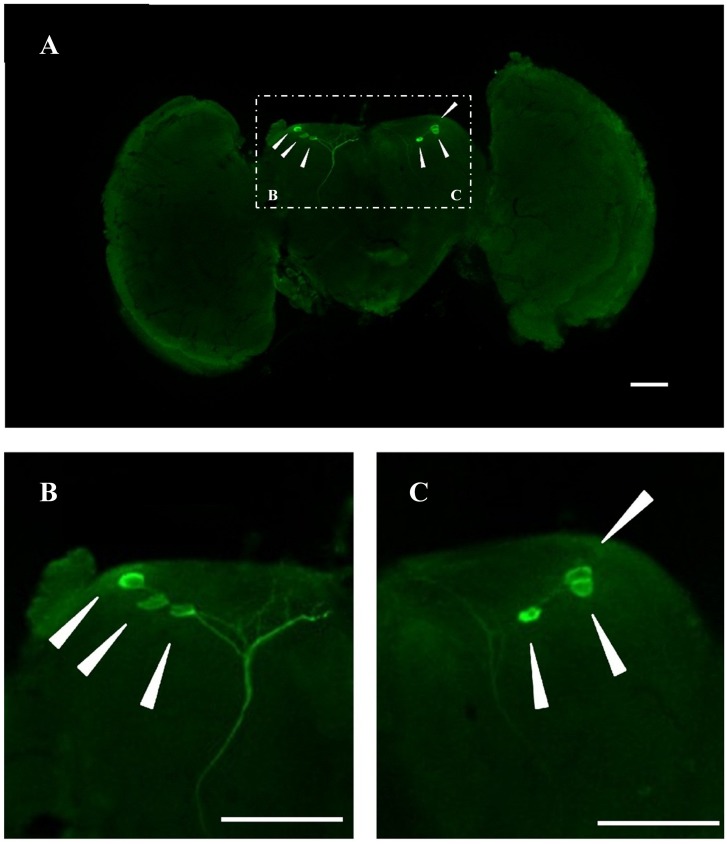
Immunoreactive *BdCrz* neurons in the brain of 9-day-old male adult of *B. dorsalis*. **(A)** Whole brains of adults stained with Crz-specific antiserum. **(B,C)** Show the stained cell body cluster at higher magnification, which are the white boxed areas in **A**. Arrows indicate where the neurons are located. Scale bars = 100 μm.

In an attempt to gain insight into the localization of *BdCrz* in the brain of male adults of *B. dorsalis*, whole mount immunohistochemistry was carried out with a rabbit antibody against *D. melanogaster* mature Crz. **Figure 2** shows a representative image of 20 different samples. As the result indicated, three pairs of adult Crz-immunoreactive neurons were observed in a cluster in the dorsolateral region of the protocerebrum (DLP).

### Knockdown of *BdCrz* or *BdCrzR* by RNAi

**FIGURE 3:**
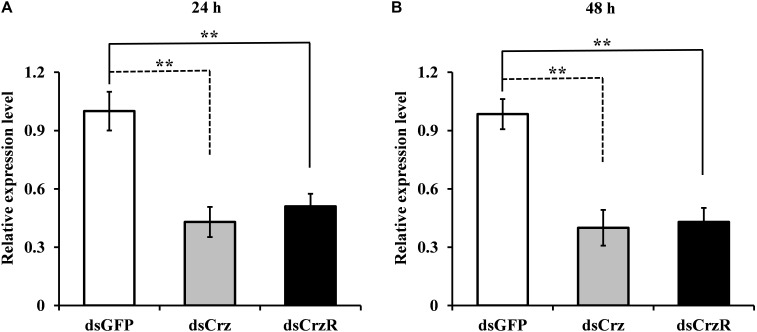
Effect of Crz or CrzR-double-stranded RNA (dsCrz or dsCrzR) injection on gene transcript levels in the 9-day-old male adult of *B. dorsalis*. Knockdown efficiency was measured at 24 h **(A)** and 48 h **(B)** after dsRNA injection. *α-Tubulin* was used as an internal reference gene. Data are means ± SE based on three independent experiments. Asterisks indicate significant differences in relative expression. ^∗∗^*P* < 0.01, *t*-test.

To better understand the function of Crz signal pathway on mating behavior, we injected specific dsRNA against *BdCrz* and *BdCrzR* in 9-day-old adult flies. The results showed that the knockdown efficiencies were significant for *BdCrz* and *BdCrzR* after the dsRNA injection. Compared with the control groups injected with dsGFP, the gene silencing efficacies for *BdCrz* mRNA levels were about 60% at 24 and 48 h post-injection of dsCrz, and these for *BdCrzR* with dsCrzR injection reached 50 and 57% at 24 and 48 h post-injection, respectively (**Figure 3**).

### Effects of *BdCrz* or *BdCrzR* Knockdown on Mating Behavior

**FIGURE 4:**
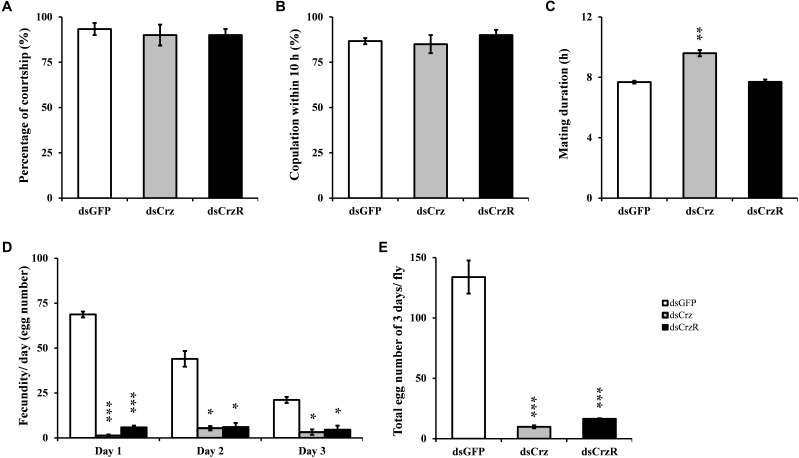
Effect of RNAi-mediated Crz or CrzR knockdown on mating behavior and fertility in the male adult of *B. dorsalis*. Proportion of couples courting **(A)**, copulation **(B)**, the corresponding mating duration **(C)** and the fecundity **(D,E)**. dsGFP-, dsCrz-, or dsCrzR-injected male was crossed with a normal female. The data are presented as means ± SE based on three independent experiments. ^∗^*p <* 0.05, ^∗∗^*p <* 0.01, ^∗∗∗^*p <* 0.001.

The effects of RNAi-mediated *BdCrz* and *BdCrzR* knockdown on mating behavior were analyzed. Consequently, the courtship behavior and mating frequency of the Crz- or CrzR-silenced males were normal as compared with the control (**Figures 4A,B**). By contrast, the silencing of Crz resulted in a dramatically increased copulation duration in males. In the control group, the copulation duration of dsGFP-treated males typically lasted 7 h, while the copulation duration of dsCrz-treated males increased with more than 40% to 10 h. Nevertheless, CrzR silencing had no effect on the copulation duration in males (**Figure 4C**). In addition, it was striking that the silencing of either Crz or CrzR caused a similar defect in fecundity: the egg-production in the respective partner female was significantly less than that in the control group (i.e., females that had mated with dsGFP-injected males) in the followed 3 days (**Figures 4D,E**). Indeed, the numbers of eggs per female after mating with a male injected with dsCrz or dsCrzR were reduced by 80–90% compared to the control.

### Effects of *BdCrz* or *BdCrzR* Knockdown on Sperm Transfer

**FIGURE 5:**
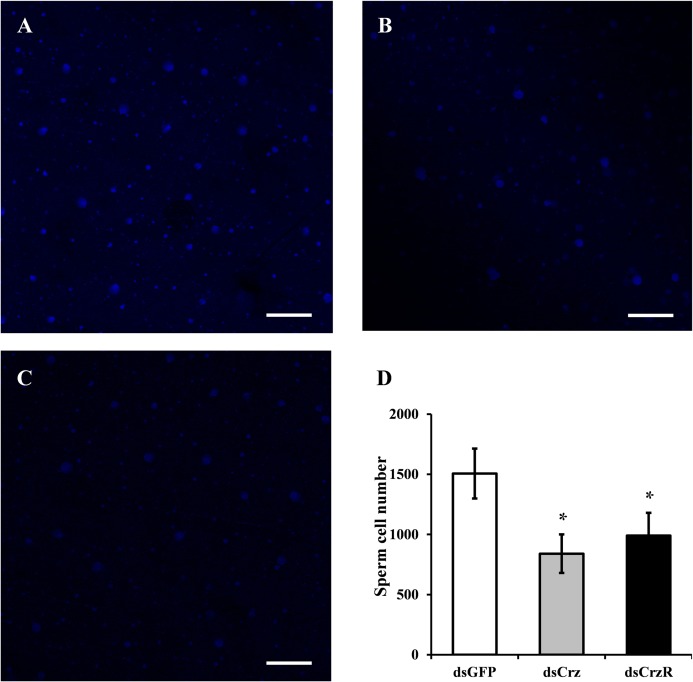
Effect on sperm transfer of *BdCrz* or *BdCrzR* knockdown in *B. dorsalis*. The DAPI staining of sperm cells in the spermatheca of mated females with a male injected with dsGFP **(A)**, dsCrz **(B)**, or dsCrzR **(C)**. Scale bars = 100 μm. **(D)** Number of sperm cells in the spermatheca of mated females with a male injected with dsGFP, dsCrz, or dsCrzR. Data are means ± SE based on three independent experiments. ^∗^*p* < 0.05, *t*-test.

To clarify the effects of Crz signaling pathway on sperm transfer in *B. dorsalis*, the reproductive organs of females, that had copulated with dsGFP-, dsCrz-, or dsCrzR-injected males, were dissected after the mating was finished and then we did a DAPI staining of sperm cells. The results showed that *BdCrz* or *BdCrzR* knockdown caused a marked lower number of sperm cells in the spermatheca compared to the control group (**Figure 5**).

## Discussion

Crz and its receptor are widespread and highly conserved in insects, but no universal function has been described so far, while its function in other basal taxa is unknown also. In the present study, we described the tissue-specific expression patterns of *BdCrz* and *BdCrzR* in the male adult of the oriental fruit fly. Furthermore, we have delineated a neural function of Crz pathway in the sexual behavior and male fertility in *B. dorsalis* by RNAi.

The tissue-specific expression analysis of *BdCrz* revealed that mRNA accumulation was greatest for the CNS of the male adult *B. dorsalis*, and no detectable qPCR products existed in other tissues. Immunohistochemistry showed that three pairs of Crz neurons are located in the DLP region of the adult CNS. These results seem to be equivalent to the data obtained in *D. melanogaster* and *Musca domestica* (Choi et al., 2005; Lee et al., 2008; Sha et al., 2012). A previous study demonstrated the production of Crz in the larval CNS of *B. dorsalis*, specifically by a group of three neurons in the dorsolateral (DL) region and eight pairs in the ventral nerve cord (VNC) (Hou et al., 2017b). In *D. melanogaster*, both the VNC and dorso-medial (DM) groups of Crz neurons of the larva die via programmed cell death during metamorphosis, whereas the DL neurons that are involved in the regulation of trehalose metabolism persist to adulthood and form a cluster of neurons, that are called DLP neurons (Choi et al., 2006; Lee et al., 2008; Kubrak et al., 2016). Holometabolous insects display substantial changes in morphology and behavior from juveniles to adults, whereas reproduction is a primary task of the adult for survival (Lee et al., 2008). Here, our results may also indicate the distinct fates of Crz neurons during CNS metamorphosis in *B. dorsalis*.

Previously, we found that *BdCrzR* are located in both the CNS and epitracheal gland in the larval stage of *B. dorsalis* (Hou et al., 2017b). Here, qPCR analysis demonstrated that *BdCrzR* transcript was most abundant in the fat body and CNS of the male adult of *B. dorsalis*. Consistent with this pattern, expression of CrzR occurred both in the heads and bodies with no sexual difference in *M. domestica* (Sha et al., 2012). In the *Drosophila* adult, high expression levels of CrzR were substantiated in the fat body, heart, and salivary glands, and a low level was determined in the CNS (Kubrak et al., 2016). In *A. aegypti* adults, quantitative spatial expression analysis revealed CrzR transcript in a variety of organs including head, thoracic ganglia, primary reproductive organs (ovary and testis), as well as male carcass (Oryan et al., 2018). These data suggest the multiple functions of the Crz signaling pathway in the adult fly. In addition, the developmental expressions of *BdCrz* and *BdCrzR* mirrored each other properly, which may indicate biologically relevant interactions between the Crz and its receptor (Hou et al., 2017b). A purpose of the present study was to test the sexual function of Crz signaling pathway in *B. dorsalis*. Our results showed that RNAi-mediated Crz knockdown lengthened the mating duration and decreased the fertility in males, and CrzR knockdown also decreased the numbers of egg in mated flies, while the behavioral sequence and the success of mating with Crz or CrzR knockdown were the same as the control flies. Injection of Crz in transitioning *H. saltator* individuals suppressed expression of vitellogenin in the brain and inhibited gamergate behaviors, such as dueling and egg deposition (Gospocic et al., 2017). Given the enrichment of CrzR in the primary and secondary sex organs of adult mosquitoes of *A. aegypti*, Crz signaling may play a role in regulating the reproductive biology (Oryan et al., 2018). In *Drosophila*, the silencing of Crz neurons resulted in infertility and extended copulation duration, and these two phenotypes were specific to males (Tayler et al., 2012). Our findings seem to be in accordance with the phenotypes obtained above. As reported by Acebes et al. (2004), copulation involves the transfer of sperm and seminal fluid, including substances that change female fertility and postmating behavior. In the present study, *BdCrz* or *BdCrzR* knockdown decreased the numbers of sperm cells in the reproductive organs (spermatheca) of the mated females, which is indicating that Crz signaling pathway modulates the reproduction via sperm transfer in *B. dorsalis*. In *Drosophila*, it has been reported that Crz controls the transfer of sperm and seminal fluid and copulation duration, thereby coupling the timing of these two processes for transfer of sperm and seminal fluid (Tayler et al., 2012).

A Crz-type peptide-receptor signaling pathway has also been identified in other protostomian invertebrates (e.g., annelids and mollusks) and in deuterostomian invertebrates (e.g., echinoderms), as demonstrated by phylogenetic analysis of genome/transcriptome sequence data and receptor deorphanization (Tian et al., 2016; Zandawala et al., 2017). In the starfish *A. rubens*, pharmacological experiments revealed that Crz causes contraction of cardiac stomach, apical muscle, and tube foot preparations, which are indicatives of its physiological roles (Tian et al., 2017). The evidence of a reproductive role has been reported in *A. californica*, where the long form of the Crz receptor is expressed in parts of the ovotestis (Kavanaugh and Tsai, 2016).

Despite the fact that the complex mechanism of how the Crz signaling system is controlling the copulation physiological and behavioral programs remains to be elucidated, the present study indicated that Crz signaling pathway in the male adult is closely related to the mating duration and reproductive physiology in *B. dorsalis*. Indeed our data showed a strong loss (>80–90%) of oviposition in the recipient female adult. In conclusion, we believe this approach may be a new target that is different from all ovicidal insecticide activities reported in the female body, useful for pest control as *B. dorsalis* via development of Crz antagonists.

## Author Contributions

Q-LH, E-HC, H-BJ, YP, J-JW, and GS conceived the study and participated in its design. Q-LH and E-HC wrote the original draft and performed all of the experiments with the help of X-QL, S-FY, and P-JY. J-JW and GS provided the materials. Q-LH, E-HC, H-BJ, and GS analyzed the data. H-BJ, YP, J-JW, and GS edited and reviewed the paper.

## Conflict of Interest Statement

The authors declare that the research was conducted in the absence of any commercial or financial relationships that could be construed as a potential conflict of interest.
